# miRNA-93-5p in exosomes derived from M2 macrophages improves lipopolysaccharide-induced podocyte apoptosis by targeting Toll-like receptor 4

**DOI:** 10.1080/21655979.2021.2023794

**Published:** 2022-03-15

**Authors:** Zhu Wang, Wansen Sun, Ruiping Li, Yan Liu

**Affiliations:** Department of Traditional Chinese Medicine, The Second Affiliated Hospital of Xi’An Jiaotong University Health Science Center, Xi’an,Shaanxi, China

**Keywords:** Diabetic nephropathy, exosome, macrophage, miR-93-5p, TLR4

## Abstract

Diabetic nephropathy (DN) is a common complication of diabetes mellitus which can result in renal failure and severely affect public health. Several studies have revealed the important role of podocyte injury in DN progression. Although, the involvement of exosomes derived from M2 macrophages has been reported in podocyte injury, the underlying molecular mechanism of M2 macrophage-secreted exosomes has not been fully elucidated. Our study suggests that M2 macrophages mitigate lipopolysaccharide (LPS)-induced injury of podocytes via exosomes. Moreover, we observed that miR-93-5p expression was markedly upregulated in exosomes from M2 macrophages. Inhibition of miR-93-5p derived from M2 macrophage exosomes resulted in apoptosis of LPS-treated podocytes. Additionally, TLR4 showed the potential to bind to miR-93-5p. Subsequently, we validated that TLR4 is a downstream target of miR-93-5p. Further findings indicated that silencing of TLR4 reversed the renoprotective effects of miR-93-5p-containing M2 macrophage exosomes on LPS-induced podocyte injury. In summary, our study demonstrated that M2 macrophage-secreted exosomes attenuated LPS-induced podocyte apoptosis by regulating the miR-93-5p/TLR4 axis, which provides a new perspective for the treatment of patients with DN.

## Introduction

Diabetic nephropathy (DN) is a serious microvascular disease secondary to diabetes mellitus and a leading contributor to end-stage renal disease worldwide [1–4]. Epidemiological data show that approximately 40% of diabetes mellitus cases progress to DN [5]. The major clinicopathological traits of DN include glomerular hypertrophy, albuminuria, enhanced extracellular matrix accumulation, thickened basement glomerular membrane, and podocyte injury [6,7]. During the progression of DN, the interaction between pro apoptotic and anti apoptotic Bcl-2 family members is unbalanced, resulting in cell death [8]. Apoptosis of renal cells, as the pathogenic and initiating mechanism of renal remodeling in DN, eventually leads to glomerular basement membrane expansion, glomerulosclerosis and tubular cell hypertrophy. At the same time, the reduction of normal renal cells caused by abnormal and excessive apoptosis is the key pathogenesis of renal fibrosis [9,10]. Therefore, paying attention to renal cell apoptosis may be the key to the treatment of DN

Podocytes, also known as glomerular visceral epithelial cells, are important elements of the renal filtration barrier and exert a critical effect in regulating the function of the glomerular filtration barrier via endocytosis, secretion of cytokines, and participation in local renal immune response [8,11–14]. Inflammatory cell infiltration is a critical pathological feature in DN development [15]. In DN patients, macrophages are the most abundant among infiltrating immune cells in the kidney, and macrophage accumulation commonly indicates renal function decay [16]. Macrophages are plastic and pluripotent and can change their phenotypes in response to different environmental signals [17,18]. According to their functions, macrophages are classified into two categories: classical proinflammatory M1 macrophages and alternative anti-inflammatory M2 macrophages [19]. Mounting evidence suggests that M2 macrophages play a inhibitory role in the progression of DN [20,21].

Exosomes are membrane-bound vesicles that are crucial mediato rs of the process of intercellular communication by delivering miRNAs, proteins, and other functional molecules to recipient cells [18,22,23]. Notably, M2 macrophage-derived exosomes are involved in the physiological and pathological progression of multiple kidney diseases, including DN [24,25]. However, the potential mechanism of M2 macrophage-derived exosomes during DN pathogenesis remains largely unknown.

In the current study, we aimed to elucidate the mechanism of action of M2 macrophages in DN development. We hypothesized that M2 macrophage-derived exosomes inhibited podocyte apoptosis by regulating the miR-93-5p/TLR4 pathway, which may provide a novel strategy for DN therapy.

## Materials and methods

### Cell culture and treatment

Conditionally immortalized mouse podocytes and macrophages RAW264.7 were supplied by Cell Bank of the Chinese academy of sciences (Shanghai, China). Podocytes were grown in RPMI 1640 (A4192301, Gibco, USA) containing 10% FBS, 100 U/ml interferon (IFN)-γ and 1% streptomycin/penicillin at 33°Cin the presence of 5% CO_2_. Macrophages were maintained in DMEM (SH30585.02, Hyclone, USA) with 10% FBS, 1% streptomycin/penicillin and cultured in a moist incubator under 5% CO_2_ at 37°C. To obtain M1 phenotype macrophages, RAW264.7 cells were stimulated by 100 ng/mL lipopolysaccharide (LPS, 916374, Sigma, USA). Meanwhile, the macrophages were induced by 20 ng/mL interleukin (IL)-4 (I4269, Sigma, USA) for M2 macrophage polarization. For establishment of DN model in vitro, podocytes were subjected to LPS treatment (100 ng/mL) for 48 h. To prevent the release of exosomes, macrophages were treated with 10 μM exosome inhibitor GW4869 (6823–69-4, Millipore, USA).

### Exosome extraction

According to a previous study [26], the exosomes were isolated from the supernatant of macrophage culture media via differential centrifugation. Briefly, for the removal of cells and debris, the harvested cell supernatants were centrifuged successively at 500 × g for 10 min, 3000 × g for 15 min, and 12,000 g for 30 min in a 4°C centrifuge. The exosomes were purified by centrifugation at 140,000 × g for 1.5 h twice.

### Transmission electron microscopy (TEM)

Exosome identification was performed by TEM as described by Yang et al [27]. Exosome samples were fixed with 2.5% buffered glutaraldehyde overnight and stained with 1% osmium tetroxide. Following gradient ethanol dehydration, the exosomes were embedded in epoxy resin and photographed using TEM.

### Cell transfection

For regulation of miR-93-5p expression level, miR-93-5p mimics, miR-93-5p inhibitors, NC mimics and NC inhibitors were designed and provided by GenePharma (Shanghai, China). To knock down or over expressed TLR4 expression, the siRNAs against TLR4 and over expressed TLR4 were obtained from Generalbio (Anhui, China) with nonspecific siRNAs and vector as negative control. The siRNAs sequences were as follows: miR-93-5p inhibitor, AGGTAGTGTGATTACCCAACCTACT; Si-TLR4, CACGGCATCTTTACTGGCTTAGTCA. Cells were plated to 6-well plates at 4 × 10^5^ cells/well and transfected with the mentioned plasmids or oligonucleotides using Lipofectamine 3000 (Thermo Fisher Scientific, USA) obeying the manufacturer’s directions.

### Flow cytometric analysis

Cell apoptosis was carried out using an Annexin V-FITC/PI apoptosis detection kit (BD Biosciences, USA) according to the previous study [28]. In brief, podocytes were washed twice with phosphate buffered saline (PBS), resuspended in 1 × Annexin binding buffer, and treated with 5 μL of FITC Annexin V and 5 μL of PI reagent in the dark. The apoptosis rate of the podocytes was determined using a flow cytometer (556570, BD Biosciences).

### Western blotting

As described by Taylor et al [29]. Total protein was isolated using radioimmuno precipitation assay (RIPA) buffer (P0013C, Beyotime, China), separated using sodium dodecyl sulfate polyacrylamide gel electrophoresis (SDS-PAGE), transferred to PVDF membranes, and sealed in 5% skimmed milk. The membranes were treated overnight with primary antibodies against CD9 (ab236630, 1:600), CD63 (ab134045, 1:800), TSG101 (ab125011, 1:1000), CD163 (ab182422, 1:500), CD206 (ab64693, 1:1200), CD68 (ab283654, 1:1000), Bax (ab32503, 1:700), cleaved caspase 3 (ab32042, 1:600), Bcl-2 (ab141523, 1:800), and GAPDH (ab8245, 1:2000) purchased from Abcam (Cambridge, USA) at 4°C. The membranes were then probed with HRP-labeled secondary antibodies at room temperature for 2 h, followed by visualization with an enhanced chemiluminescence (ECL) kit and image analysis using ImageJ software. GAPDH was used as an internal control.

### Co-culture of podocytes with macrophages and exosomes

According to a previous study [30], Macrophages were seeded in the bottom of a Transwell chamber with 0.4 μm pore size membrane and podocytes were placed in the upper chamber. The podocytes were collected for subsequent experiments after 24 h of co-culture. To assess the effects of exosomes, the podocytes were treated with exosomes extracted from macrophages for 24 h and analyzed by flow cytometry and Western blotting.

### Quantitative realtime PCR (qRT-PCR)

According to a previous study [31], total RNA was isolated using TRIzol® reagent (T9424, Invitrogen, USA). The PrimeScript RT kit (6215A, Takara, Japan) was used for cDNA synthesis. Gene expression was detected by PCR in a LightCycler PCR system (Bio-Rad, USA) using SYBR® Green Master Mix II (RR036Q, Takara) according to the manufacturer’s instructions.

### PKH67-labeled exosome transfer assay

According to a previous study [32], to identify the cellular uptake of exosome, PKH67-marked exosomes from M2 macrophages were employed. PKH67 green fluorescent linker Mini Kit (MINI67, Sigma, USA) was used for the label according to the manufacturer’s instructions.

### Dual-luciferase reporter assay

Wild-type (WT) TLR4 containing miR-93-5p binding sites and mutant (MUT) TLR4 without miR-93-5p binding sites were inserted into the psiCHECK-2 plasmid (Promega, USA) to generate TLR4-WT and TLR4-MUT constructs. Using Lipofectamine™ 3000 (Thermo Fisher Scientific), podocytes were co-transfected with the indicated reporter vectors and miR-93-5p mimics or NC mimics, respectively. Luciferase activity was measured using a Dual-Luciferase® Reporter Assay System (E1910, Promega) 24 h after transfection following the manufacturer’s protocols.

### Statistical analysis

All experimental results (from three independent assays) are expressed as the mean ± standard deviation. Data processing was performed using GraphPad Prism 6.0. Comparisons between two or more groups were analyzed using the Student’s *t*-test or one-way ANOVA. Differences were considered statistically significant at *P *< 0.05.

## Results

This study demonstrated that miR-93-5p expressions were up-regulated in M2 macrophage-secreted exosomes. After co-culturing of podocytes with macrophages and exosomes, we confirmed that M2 macrophage-secreted exosomes alleviate LPS-induced podocyte injury. Mechanistically, miR-93-5p in exosomes from M2 macrophages restrained the apoptosis of LPS-induced podocytes by regulating the miR-93-5p/TLR4 pathway.

### M2 macrophages inhibit apoptosis of LPS-induced podocytes by secreting exosomes

**Figure 1.**
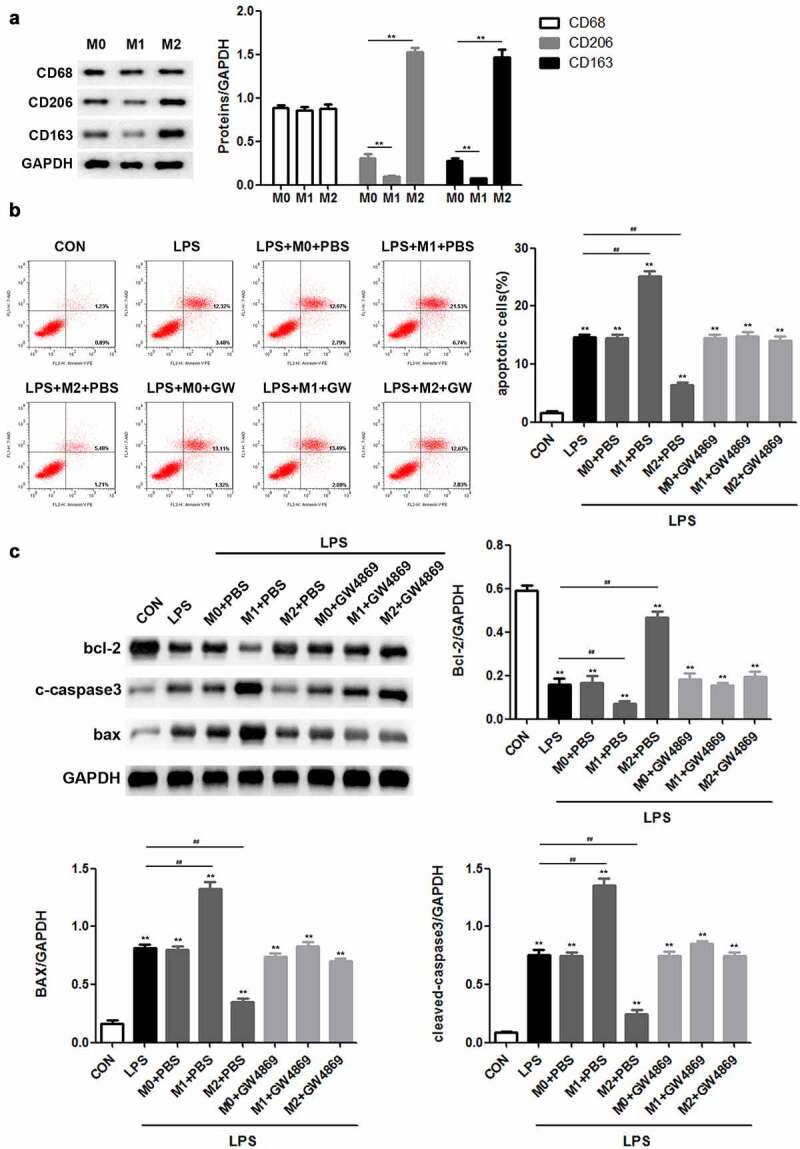
M2 macrophages inhibit apoptosis of LPS-induced podocytes through secreting exosomes. (a) Western blot analysis of CD68, CD206 and CD163 expression levels. (b) Flow cytometric analysis was carried out to ascertain the mode of action of macrophages in podocyte injury. (c) After LPS, M0, M1, M2 and GW4869 treatments, the expression of Bax, Bax, and cleaved caspase 3 in podocytes was detected by Western blotting. LPS, lipopolysaccharide; M0, M1, M2, different subtypes macrophage. **P < 0.01.

First, we sought to investigate the role of macrophages in LPS-induced podocyte injury. Western blotting was performed to determine the expression of major biomarkers in the different macrophage subtypes. The results revealed that there was no obvious difference in the levels of macrophage marker CD68 among M0, M1, and M2 macrophages, whereas CD206 and CD163 were highly expressed in M2 macrophages (Figure 1(a)). Flow cytometric analysis demonstrated that LPS stimulation promoted podocyte apoptosis. M1 macrophages exacerbated the apoptosis of LPS-induced podocytes, whereas M2 macrophages caused the opposite result. Notably, GW4869, an inhibitor of exosome release, inhibited the function of M1 and M2 macrophages in LPS-induced podocyte apoptosis (Figure 1(b)). Consistently, M1 macrophages enhanced LPS-induced upregulation of Bax and cleaved caspase 3 expression as well as downregulated the level of Bcl-2, whereas M2 macrophages resulted in the recovery of the expression levels of the indicated proteins. It is likely that GW4869 counteracted the effects of macrophages on Bcl-2, Bax, and cleaved caspase 3 expression (Figure 1(c)). Taken together, these data provide strong evidence that M1 and M2 macrophages act as regulators of LPS-induced podocyte injury by secreting exosomes.

### M2 macrophage-derived exosomes alleviate injury of LPS-treated podocytes

**Figure 2.**
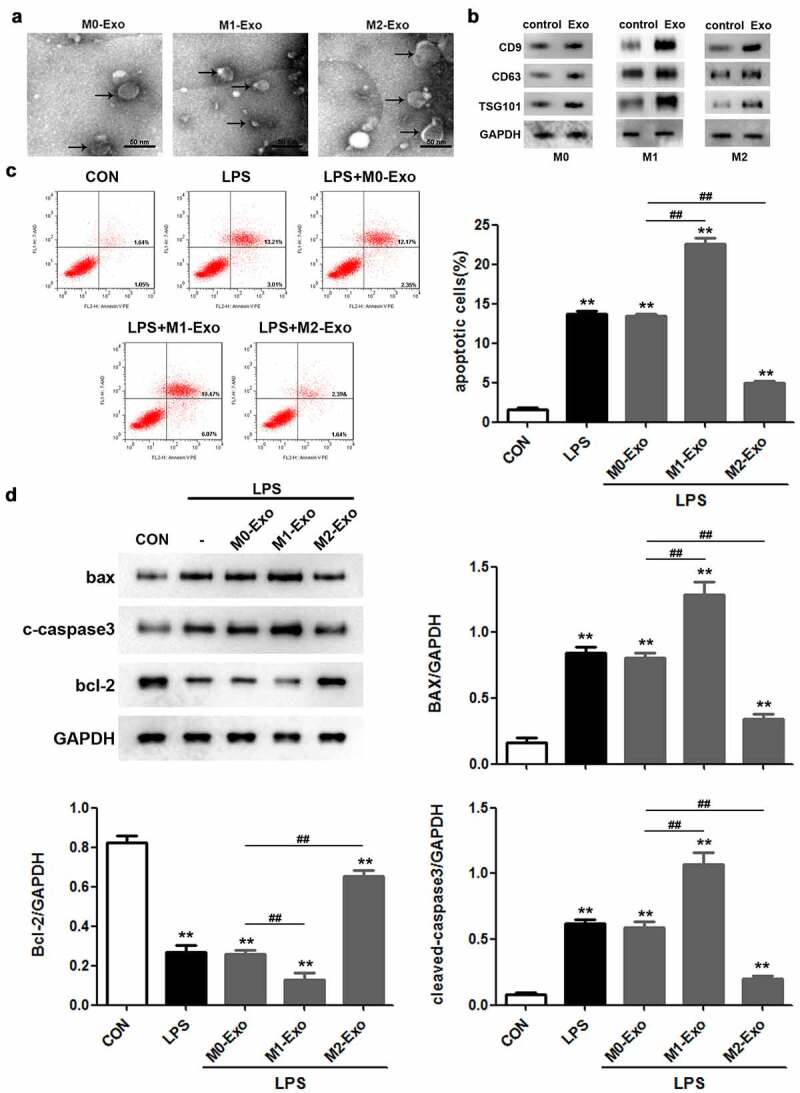
M2 macrophage-derived exosomes alleviate the injury of LPS-treated podocytes. (a) Exosomes isolated from different macrophage subtypes were identified with transmission electron microscopy (TEM). (b) Western blot analysis of CD9, CD63 and TSG101 levels in macrophage-derived exosomes. (c) Apoptosis of podocytes determined by flow cytometric analysis. (d) Western blotting was performed to detect the expression of Bcl-2, Bax and cleaved caspase 3 in podocytes. LPS, lipopolysaccharide; Bcl-2, B-cell lymphoma-2; Bax, BCL2-Associated X; **P < 0.01 vs CON, ^##^*P* < 0.01.

To identify the potential involvement of exosomes secreted by macrophages in LPS-induced podocyte injury, exosomes were obtained from the culture supernatant of macrophages. TEM images showed that the exosomes were solid and dense (Figure 2(a)). Western blot analysis indicated that the levels of the important exosome markers including CD9, CD63, and TSG101 were markedly increased in the exosomes compared to the control group, confirming the successful isolation of exosomes (Figure 2(b)). Flow cytometric analysis revealed that LPS triggered a significant increase in the apoptosis rate of podocytes, and M1 macrophage-derived exosomes further promoted LPS-induced podocyte apoptosis, whereas exosomes secreted by M2 macrophages attenuated the apoptosis of LPS-treated podocytes (Figure 2(c)). Similarly, we observed that co-culture of M1 macrophage-derived exosomes and podocytes aggravated the increased Bax and cleaved caspase 3 levels and decreased Bcl-2 expression in podocytes caused by LPS stimulation (Figure 2(d)). Based on the above findings, we concluded that M2 macrophage-derived exosomes suppress apoptosis of LPS-induced podocytes.

### miR-93-5p is highly expressed in M2 macrophage-derived exosomes

**Figure 3.**
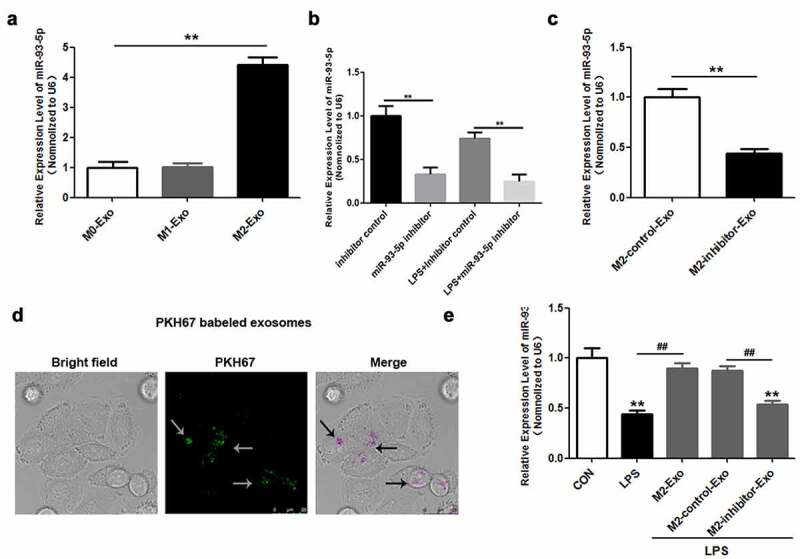
miR-93-5p is highly expressed in exosomes secreted by M2 macrophages. (a) Expression of miR-93-5p in exosomes from different macrophage subtypes was measured by RT-qPCR. (b) Transfection efficiency of miR-93-5p inhibitor was verified in macrophages treated with and without LPS by RT-qPCR analysis. (c) RT-qPCR analysis of miR-93-5p expression in exosomes transfected with miR-93-5p inhibitors. (d) Uptake of exosomal miR-93-5p into podocytes was visualized with a fluorescent microscope. (e) Following LPS, M2-Exo and miR-93-5p inhibitor treatments, miR-93-5p expression in podocytes was examined with RT-qPCR assay. LPS, lipopolysaccharide; M2, macrophage; Exo, exosomes **P < 0.01 vs CON, ^##^*P* < 0.01.

Subsequently, we sought to analyze the latent mechanism underlying the effect of macrophage-derived exosomes on LPS-induced podocyte apoptosis. miR-93-5p expression was only slightly augmented in exosomes secreted by M2 macrophages (Figure 3(a)). Accordingly, we explored the role of miR-93-5p in the effects of M2 macrophage-derived exosomes on LPS-induced podocyte injury. miR-93-5p inhibitors and NC inhibitors were used to downregulate miR-93-5p in M2 macrophages and exosomes. RT-qPCR assay showed that the expression of miR-93-5p was remarkably reduced in M2 macrophages transfected with miR-93-5p inhibitors compared to those transfected with NC inhibitors whether or not treated with LPS (Figure 3(b)). Moreover, miR-93-5p inhibitors suppressed miR-93-5p expression in exosomes extracted from M2 macrophages (Figure 3(c)). To explore whether podocytes internalized M2 macrophage-derived exosomes, we used PKH67-labeled exosomes. Figure 3(d) shows the PKH67-labeled exosomes (green) can enter podocytes (purple). Additionally, we demonstrated that exosomes secreted by M2 macrophages reversed the LPS-induced downregulation of miR-93-5p in podocytes, and inhibition of miR-93-5p abrogated the effects of M2 macrophage-derived exosomes on miR-93-5p expression (Figure 3(e)). Overall, miR-93-5p was upregulated in exosomes derived from M2 macrophages, and miR-93-5p may thus play a critical role in LPS-induced podocyte injury.

### MiR-93-5p in M2 macrophage-derived exosomes inhibited LPS-induced podocyte apoptosis

**Figure 4.**
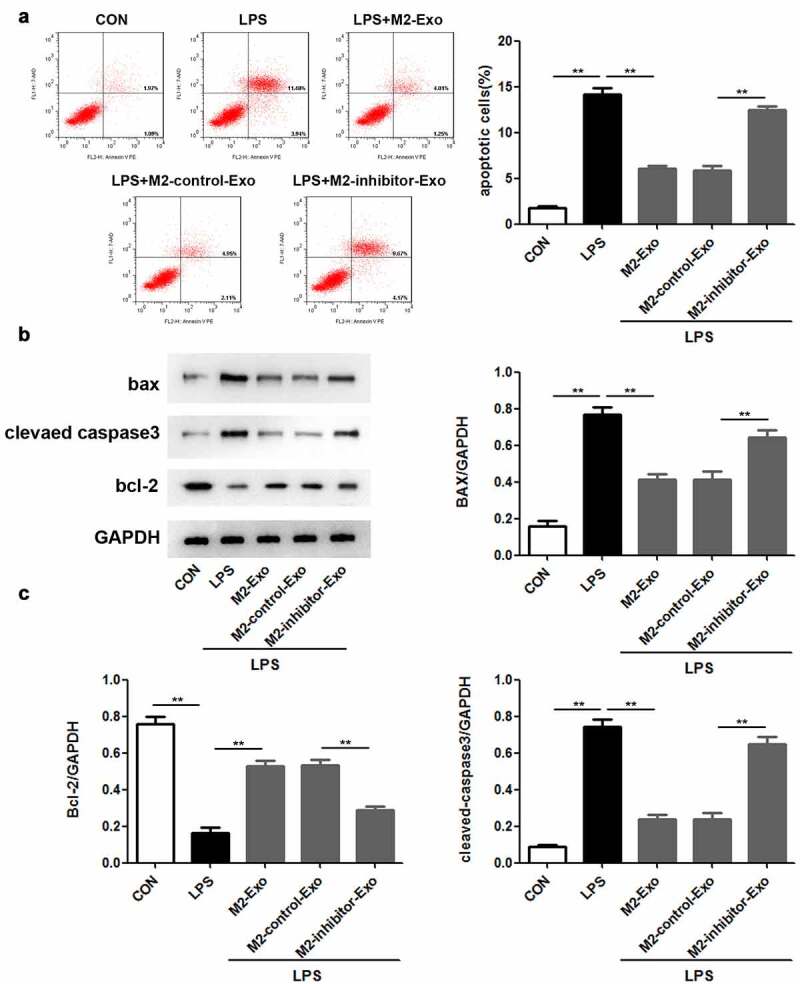
Suppression of miR-93-5p in M2 macrophage-derived exosomes facilitates LPS-induced podocyte apoptosis. (a) Apoptosis of podocytes in different groups was evaluated by flow cytometric analysis. (b) Western blotting was performed to detect the expression of Bcl-2, Bax, and cleaved caspase 3 in podocytes after LPS, M2-Exo and miR-93-5p inhibitor treatments. (c) Quantification of Bcl-2, Bax, and cleaved caspase 3 protein levels in podocytes from different groups. LPS, lipopolysaccharide; Bcl-2, B-cell lymphoma-2; Bax, BCL2-Associated X; **P < 0.01.

Next, we attempted to identify the function of miR-93-5p in LPS-induced podocyte apoptosis caused by LPS stimulation. Our observations revealed that inhibition of miR-93-5p abolished the suppressive role of M2 macrophage-secreted exosomes in the apoptosis of LPS-treated podocytes (Figure 4(a)). In agreement with the above results, Western blotting showed that the inhibition of LPS-induced upregulation of Bax and cleaved caspase-3 and downregulation of Bcl-2 in M2 macrophage-derived exosomes was reversed by miR-93-5p inhibitors (Figure 4(b-c)). Generally, M2 macrophage-secreted exosomes play a role in LPS-induced podocyte apoptosis via miR-93-5p contained in them.

### TLR4 serves as a target of miR-93-5p

**Figure 5.**
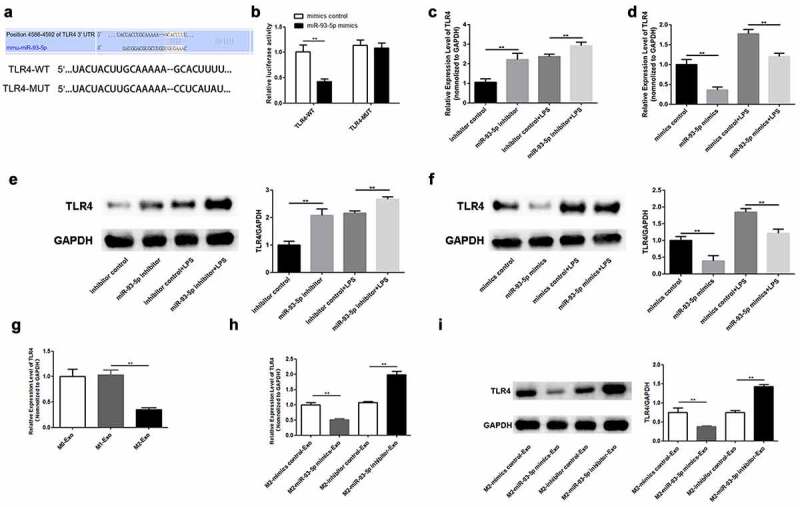
TLR4 serves as a target of miR-93-5p. (a) The predicted binding sites between miR-93-5p and TLR4. (b) Dual-luciferase reporter assay was conducted to confirm the relationship between miR-93-5p and TLR4. (c) RT-qPCR analysis of TLR4 expression in M2 macrophages transfected with corresponding oligonucleotides. (d) Western blot analysis of TLR4 protein level in M2 macrophages from different groups. (e) TLR4 expression in macrophage-derived exosomes was detected by RT-qPCR assay. (f-g) RT-qPCR and Western blot analyses of TLR4 level in podocytes treated with exosomes from transfected M2 macrophages. TLR4, Toll-like receptor 4; **P < 0.01.

Using a bioinformatics tool, we investigated the molecular regulatory mechanisms of miR-93-5p. As shown in Figure 5(a), TLR4 harbors the predicted binding sites of miR-93-5p (Figure 5(a)). Therefore, luciferase assay was conducted to verify the binding between miR-93-5p and TLR4. Our findings indicated that the luciferase activity of TLR4-WT was only reduced in the presence of miR-93-5p mimics, whereas the luciferase activity of the mutant forms showed no significant alteration (Figure 5(b)). Results of RT-qPCR and Western blotting showed that whether macrophages are treated with LPS or not, overexpression of miR-93-5p weakened the mRNA expression and protein levels of TLR4 in M2 macrophages and down-regulation of miR-93-5p abolished the suppression of TLR4 expression (Figure 5(c-f)). Furthermore, TLR4 was expressed at low levels in M2 macrophage-derived exosomes (Figure 5(g)). In agreement with these findings, we observed that TLR4 expression was decreased in podocytes co-cultured with exosomes extracted from M2 macrophages treated with miR-93-5p mimics, and that repression of miR-93-5p led to an increase in TLR4 mRNA and protein levels in podocytes treated with M2 macrophage-derived exosomes (Figure 5(h-i)). In summary, TLR4 is a downstream effector of miR-93-5p.

### M2 macrophage-derived exosomes execute a regulatory role in LPS-induced podocyte injury via miR-93-5p-mediated TLR4 expression

**Figure 6.**
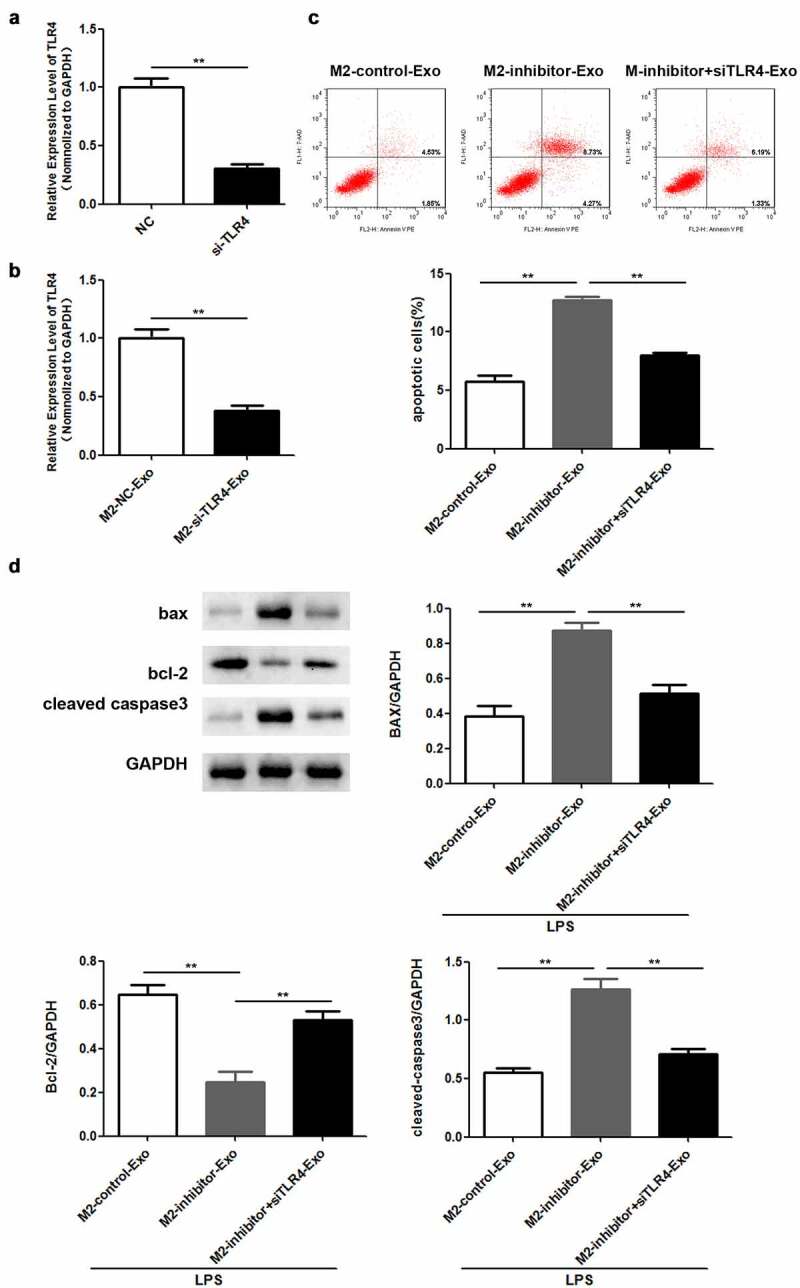
Knockdown of TLR4 reversed the role of miR-93-5p inhibitor in LPS-induced podocyte. (a) RT-qPCR was performed to measure the expression of TLR4 in podocytes transfected with si-TLR4 or negative control si-NC. (b) RT-qPCR analysis of TLR4 expression in transfected podocytes treated with exosomes from M2 macrophages. (c) Apoptosis of podocytes determined by flow cytometric analysis. (d) Protein levels of Bcl-2, Bax, and cleaved caspase 3 in podocytes following different treatments. **P < 0.01.

Lastly, we validated whether the function of exosomal miR-93-5p was mediated by TLR4. RT-qPCR analysis verified that TLR4 was downregulated in podocytes transfected with si-TLR4 (Figure 6(a)). Similarly, TLR4 was weakly expressed in podocytes co-cultured with exosomes secreted by M2 macrophages (Figure 6(b)). Flow cytometry results revealed that the apoptosis rate increased by miR-93-5p inhibitors was restored following by depletion of TLR4 in podocytes co-cultured with M2 macrophage-derived exosomes (Figure 6(c)). Western blot analysis showed that the elevated Bax and cleaved caspase 3 expression and decreased Bcl-2 levels caused by suppression of miR-93-5p were restored when TLR4 was knocked down (Figure 6(d)).

**Figure 7.**
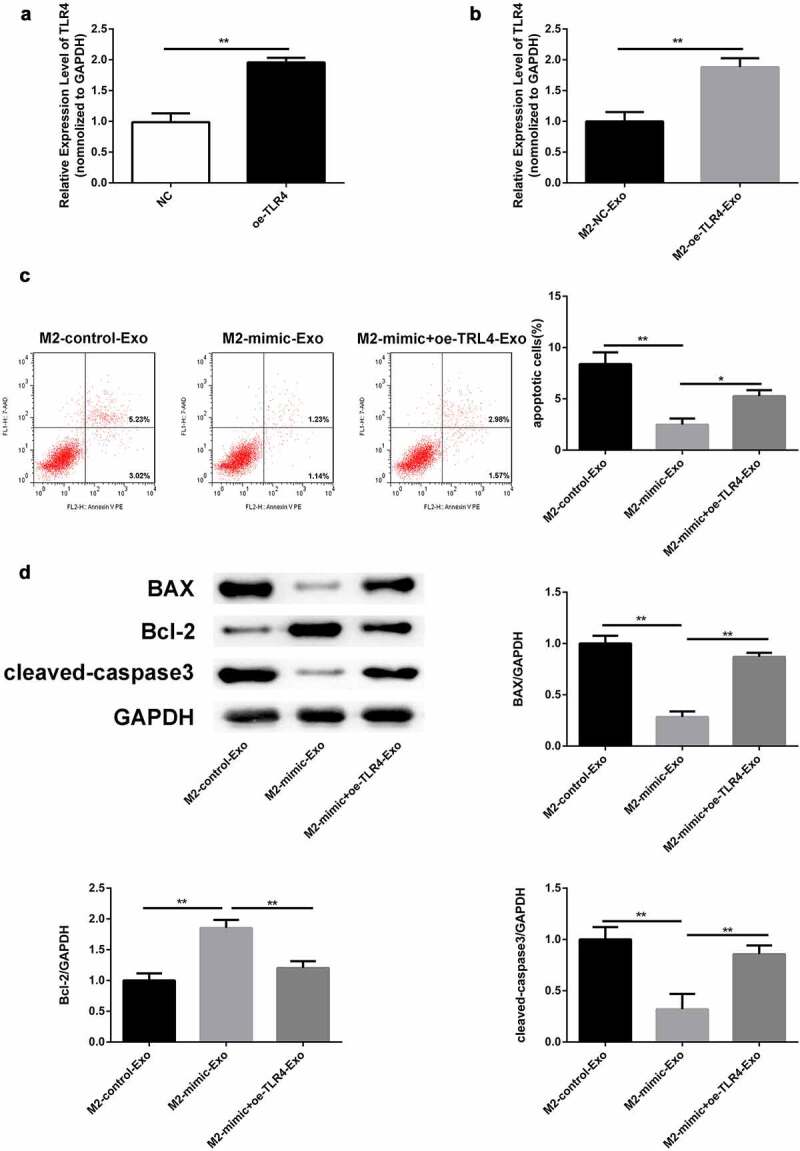
Overexpression of TLR4 reversed the role of miR-93-5p mimic in LPS-induced podocyte. (a) RT-qPCR was performed to measure the expression of TLR4 in podocytes transfected with oe-TLR4 or negative control oe-NC. (b) RT-qPCR analysis of TLR4 expression in transfected podocytes treated with exosomes from M2 macrophages. (c) Apoptosis of podocytes determined by flow cytometric analysis. (d) Protein levels of Bcl-2, Bax, and cleaved caspase 3 in podocytes following different treatments. *P< 0.05, **P < 0.01.

In addition, RT-qPCR analysis verified that TLR4 was upregulated in podocytes transfected with oe-TLR4 (Figure 7(a)). Similarly, TLR4 was over expressed in podocytes co-cultured with exosomes secreted by M2 macrophages (Figure 7(b)). Flow cytometry results revealed that the apoptosis rate decreased by miR-93-5p mimics was restored following by overexpression of TLR4 in podocytes co-cultured with M2 macrophage-derived exosomes (Figure 7(c)). Western blot analysis showed that the decreased Bax and cleaved caspase 3 expression and elevated Bcl-2 levels caused by overexpression of miR-93-5p were restored when TLR4 was up regulated (Figure 7(d)). In summary, M2 macrophage-secreted exosomes improved LPS-induced podocyte injury by targeting the miR-93-5p/TLR4 axis.

## Discussion

A wealth of data suggests that podocyte injury acts as the main determinant of the onset and evolution of DN [33–36]. Therefore, podocyte injury has become the focus of research to clarify the pathogenesis of DN in recent years [37,38]. Consequently, our study aimed to decipher the underlying molecular mechanisms of podocyte injury in DN progression. Macrophage aggregation is a significant indicator of renal insufficiency in patients with DN [39–41]. Growing evidence has expounded the protective role of M2 macrophages in podocyte injury in DN [42–44]. In the present study, we found that M2 macrophages exerted renoprotective effects on LPS-induced podocyte injury by secreting exosomes.

Macrophage-derived exosomes containing biological molecules have been shown to be involved in podocyte injury during DN progression [6,45,46]. In agreement with these results, we found that exosomes derived from M2 macrophages suppressed the apoptosis of LPS-induced podocytes. Several reports have shown that exosomal miRNAs perform crucial regulatory functions in podocyte damage [23,47]. For example, miR-25-3p in exosomes from adipose-derived stem cells attenuates high glucose-induced podocyte injury by downregulating ZEB2 expression [48]. MiRNA-16-5p secreted by human urine-derived stem cells improves DN by inhibiting podocytic apoptosis [49]. A previous study revealed that miR-93-5p was one of the most upregulated miRNAs among the differentially expressed miRNAs between M1 and M2 exosomes [24]. Consistently, our findings showed that miR-93-5p levels were higher in M2 macrophage-derived exosomes than in M1 macrophage-derived exosomes. Furthermore, miR-93-5p exhibits a protective role against a variety of cell damage caused by LPS [49–53]. Therefore, we hypothesized that miR-93-5p may ameliorate LPS-induced podocyte injury in DN. As expected, we demonstrated that repression of miR-93-5p aggravated the apoptosis of LPS-treated podocytes.

Multiple lines of evidence indicate that TLR4 plays a vital role in podocyte injury [54–56]. Using a bioinformatics database, we found that TLR4 possessed the predicted miR-93-5p binding sites. Accordingly, we selected TLR4 as the subsequent focus of our experiment. Our results validated that miR-93-5p negatively modulates TLR4 expression by binding to it. Moreover, TLR4 was found to be responsible for the function of M2 macrophage-derived exosomal miR-93-5p in LPS-induced podocyte injury.

However, there are still some limitations in this study. This study confirmed the role of M2-Exo in DN through in vitro experiments, which needs to be further verified in combination with in vivo experiments. Additionally, this study selected LPS-induced podocyte to establish a DN model, we will use other cell lines to further demonstrate our results in the future.

## Conclusion

This study revealed that M2 macrophage-secreted exosomes alleviate LPS-induced podocyte injury via miR-93-5p. Mechanistically, miR-93-5p in exosomes from M2 macrophages restrained the apoptosis of LPS-induced podocytes by regulating the miR-93-5p/TLR4 pathway, which offers novel insights into the treatment of DN.

## Highlight

- M2 macrophages inhibited the apoptosis of LPS-induced podocytes
- miR-93-5p was highly expressed in exosomes secreted by M2 macrophages
- TLR4 served as a target of miR-93-5p
